# The value of prognostic ultrasound features of breast cancer in different molecular subtypes with a focus on triple negative disease

**DOI:** 10.1007/s12282-021-01311-3

**Published:** 2021-11-15

**Authors:** Andy Evans, Yee Ting Sim, Brooke Lawson, Jane Macaskill, Lee Jordan, Alastair Thompson

**Affiliations:** 1 Mail Box 4 Ninewells Medical School, University of Dundee, Dundee, DD1 9SY USA; 2 Breast Unit, Ninewells Hospital, Dundee, DD1 9SY USA; 3 Baylor College of Medicine, 1 Baylor Plaza, Houston, TX 77030 USA

**Keywords:** Breast cancer, Ultrasound, Molecular subtype, Prognosis, Shear wave elastography

## Abstract

The ultrasound (US) features of breast cancer have recently been shown to have prognostic significance. We aim to assess these features according to molecular subtype. 1140 consecutive US visible invasive breast cancers had US size and mean stiffness by shearwave elastography (SWE) recorded prospectively. Skin thickening (> 2.5 mm) overlying the cancer on US and the presence of posterior echo enhancement were assessed retrospectively while blinded to outcomes. Cancers were classified as luminal, triple negative (TN) or HER2 + ve based on immunohistochemistry and florescent in-situ hybridization. The relationship between US parameters and breast cancer specific survival (BCSS) was ascertained using Kaplan–Meier survival curves and ROC analysis. At median follow-up 6.3 year, there were 117 breast cancer (10%) and 132 non-breast deaths (12%). US size was significantly associated with BCSS all groups (area under the curve (AUC) 0.74 in luminal cancers, 0.64 for TN and 0.65 for HER2 + ve cancers). US skin thickening was associated most strongly with poor prognosis in TN cancers (53% vs. 80% 6 year survival, *p* = 0.0004). Posterior echo enhancement was associated with a poor BCSS in TN cancers (63% vs. 82% 6 year survival, *p* = 0.02). Mean stiffness at SWE was prognostic in the luminal and HER2 positive groups (AUC 0.69 and 0.63, respectively). In the subgroup of patients with TN cancers receiving neo-adjuvant chemotherapy posterior enhancement and skin thickening were not associated with response. US skin thickening is a poor prognostic indicator is all 3 subtypes studied, while posterior enhancement was associated with poor outcome in TN cancers

## Introduction

Patients with invasive breast cancer are increasing likely to receive neoadjuvant systemic therapy than in previous years [1]. As such patients do not receive immediate surgery, the surgical specimen which has usually been used for prognostic, assessment [2] is not available. The only histological information available is from the core biopsy sample. As a consequence, traditional prognostic information is not available in many women currently presenting with breast cancer.

However, recent work has highlighted prognostic indicators which are available pre-operatively in almost all patients. A number of the ultrasound (US) features of breast cancer have recently been shown to indicate a poor outcome. These include large tumour size [3], skin thickening at the tumour site and posterior echo enhancement [4]. Skin thickening on US and distance of the tumour from the skin have also previously been shown to correlate with the presence of nodal metastases and axillary nodal burden in those women undergoing immediate axillary clearance. Posterior echo enhancement has previously been shown to correlate with high histological grade and triple negative disease. Peri-lesion stiffness on shear wave elastography (SWE) which is an ultrasound technique has recently been shown to be an independently associated with breast cancer death. Stiffness at elastography is thought to represent abnormal collagen structure in activated peri-tumoural stroma [5, 6]. Skin thickening and peri-lesional stiffness have previously been shown to have good intra-observer reproducibility [7, 8]. The reproducibility of classifying the presence or absence posterior echo enhancement is unknown.

The differing prognosis and response to neoadjuvant chemotherapy of the different molecular subtypes of breast cancer is well known and well reported [9]. Clinically useful prognostic indicators for women with luminal node negative cancers include a number of commercially available platforms including Oncotype DX [10]. Such tests are, however, expensive and time consuming. However, similar tests are not available for triple negative cancers as putative markers such as androgen receptor and stromal tumour infiltrating lymphocytes (TIL’s) have either not gained widespread acceptance or have produced conflicting results [11, 12].

As US is performed on nearly all breast cancer patients and images are easily reviewed, any prognostic information derived from these images could be freely and readily available. The aim of this study is, therefore, to assess, in different molecular subtypes, the value of established prognostic ultrasound (US) features of breast cancer.

## Materials and methods

The study was carried out in a single breast unit which sees patients with symptoms and those with abnormal screening mammography.

US lesion size, mean stiffness of the cancer (kPa) at SWE and immunohistochemical data (Oestrogen receptor (ER), Progesterone receptor (PR), and HER-2 status) were collected prospectively from a consecutive series of patients undergoing diagnostic breast US examination for lesions subsequently shown to be invasive breast cancer (*n *= 1141). These patients presented between April 2010 and September 2015. Patients diagnosed with metastatic disease at presentation were excluded. US lesion size measured both the hypoechoic and centre and the hyperechoic halo.

Patients were included irrespective of subsequent treatment (primary surgery, neoadjuvant systemic therapy and primary endocrine therapy). All US scans were performed by one of six operators trained to perform and interpret breast ultrasound using a 12 MHz probe. These practitioners had between 7 and 22 years of breast ultrasound experience and had at least 12 months of experience performing breast SWE. Four SWE images in two orthogonal planes were obtained. A 2 mm region of interest (ROI) was used. Mean stiffness in kPa was taken as the average of the values taken from four SWE images. The maximum US diameter used in the analysis was the largest obtained in any of the three planes. All scans were performed using an Aixplorer® ultrasound system (SuperSonic Imagine, Aix en Provence, France).

**Fig. 1:**
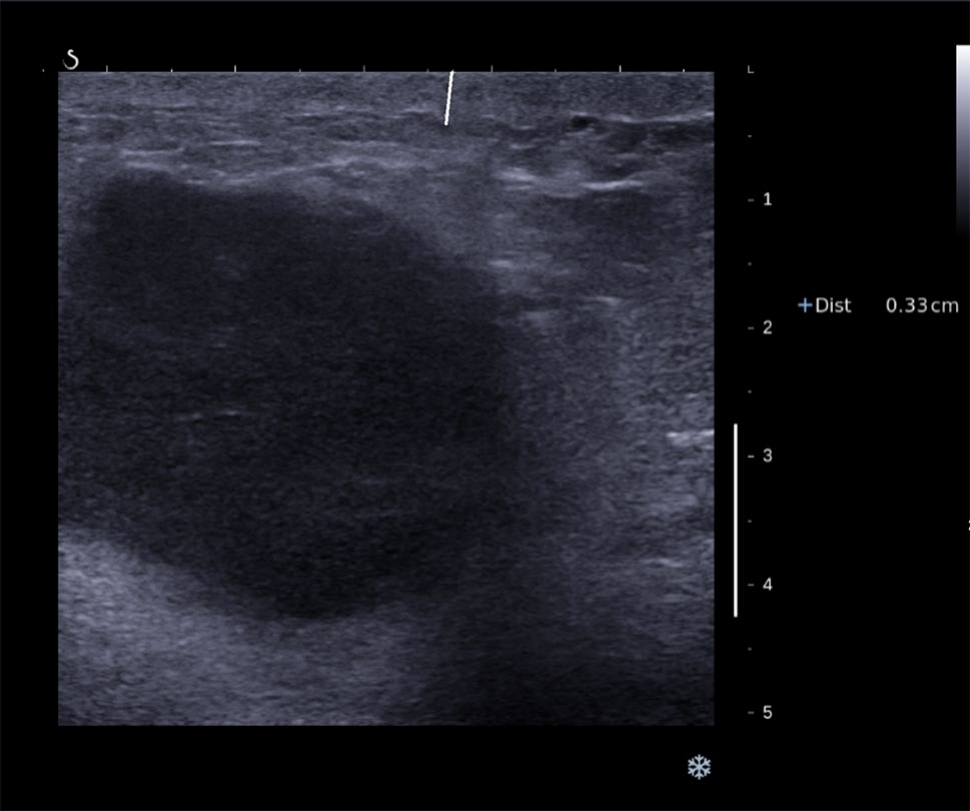
Ultrasound image of a triple negative breast cancer showing thickening of the overlying skin as indicated by the callipers

**Fig. 2:**
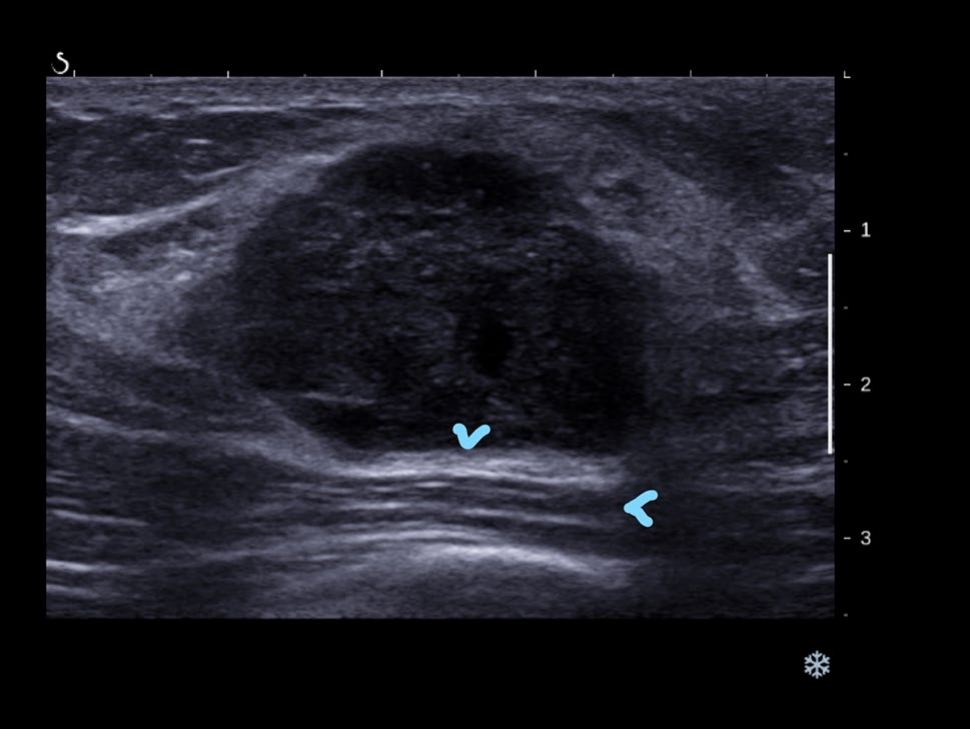
Ultrasound image of a triple negative breast cancer showing posterior echo enhancement as shown by the arrow heads

The presence of overlying skin thickening (> 2.5 mm) at US (Fig. 1) and the presence of posterior echo enhancement (Fig. 2) (features recently found to be prognostic in women with invasive breast cancer) were assessed retrospectively while blinded to outcomes. The skin thickening cutoff of 2.5 mm was decided upon, because normal breast skin measures up to 2 mm so a 2.5 mm cutoff would avoid diagnosing women with skin thickening who were at the upper border of the normal range. Patient’s survival including cause of death was ascertained from local paper and electronic health records. Patients that died after developing metastatic breast cancer were assumed to have died of breast cancer.

Cases were classified as luminal, triple negative (TN) and HER2 + ve on the basis of immunohistochemistry and florescent in-situ hybridization. Ki67 was not routinely available so splitting the luminal group into A and B was not possible. Patients who were HER-2 positive and ER positive were included in the HER-2 positive group. Core biopsy estimated histological grade is given rather than final histological grade as this can be used in all patients, however, treated. Cause and date of death were ascertained from local electronic databases.

The reproducibility of categorisation of US posterior echo enhancement was performed on a subset of 154 patients in whom two radiologists independently classified the presence or absence of posterior echo enhancement.

A sub-analysis of the TN cancers who underwent neoadjuvant chemotherapy (NACT) was performed to assess associations between responses to NACT as measured by residual cancer burden (RCB) scores and those US features found to be prognostic.

Statistical analysis was performed using Med Calc^®^ (MedCalc Software Ltd., Ostend, Belgium).

.The relationship between US parameters and breast cancer specific survival (BCSS) was ascertained using Kaplan–Meier survival curves and ROC analysis for the three subgroups. The difference between Kaplan–Meier curves was assessed using the log-rank test and the difference between ROC curves was assessed using the DeLong method. Agreement on the presence of posterior echo enhancement was assessed using Kappa statistics.

## Results

1141 patients constituted the study group (mean age 62.7 years, median age 62.6 years and range 24–95 years). 433 (38%) patients had their cancer diagnosed at mammographic screening, while 708 (62%) women had symptomatic cancers. Mean follow-up in those alive was 6.3 years. During follow-up 117 breast cancer and 134 non-breast cancer deaths occurred.

The number of cancers and breast cancer deaths in each group were; luminal *n* = 857 (75%) and 54 (6%), TN *n* = 143 (12.7%) and 36 (25%), and HER2 + ve *n* = 141(12.3%) and 27 (20%). The pathological details and systemic therapy given of the entire cohort and subgroups are shown in Table 1. Invasive size and nodal status is only given for those treated with immediate surgery. Micro-metastases and isolated tumour cells were counted as node negative. The frequency of categorical variables by subtype were: posterior enhancement, TN 41%, HER2 + ve 21% and luminal 11%. Skin thickening TN % 25%, HER2 + ve21% and luminal 12%. Mean US size and stiffness for TN, HER2 + ve and luminal subtypes were 22 mm, 22 mm and 16 mm and 137 kPa, 141 kPa and 126 kPa, respectively.

**Table 1:** Pathological and treatment details of the subgroups

Pathological feature	Whole cohort number (%)	Luminal number (%)	HER2 positive number (%)	Triple negative number (%)
Core biopsy estimated Histological grade 1	139 (12%)	136(16%)	2(1%)	1(1%)
Core biopsy estimated Histological grade 2	622(55%)	555(65%)	44(31%)	23(16%)
Core biopsy estimated Histological grade 3	380(33%)	166(19%)	95(67%)	119(83%)
Node positive	248 of 893 (28%)	189 of 715 (26%)	37 of 89 (42%)	22 of 89 (25%)
Invasive size > 20 mm	367 of 899 (40%)	271 of 718 (38%)	48 of 88 (55%)	48 of 93 (52%)
Neoadjuvant chemotherapy	120(10%)	31(4%)	42(30%)	47(33%)
Neoadjuvant/adjuvant chemotherapy	366(32%)	186(22%)	92(65%)	88(62%)
Adjuvant or neoadjuvant endocrine therapy	911(80%)	811(95%)	100(71%)	0
Total in each subgroup	1141	857(75%)	141(12%)	143(13%)

The relationship between BCSS and US features in subgroups are summarised in Tables 2 and 3 including hazard ratios (HR) and 95% confidence intervals (CI) for categorical variables.

**Table 2:** Association between continuous US features and breast cancer specific survival in women by breast cancer subtype

US feature	Subtype	AUC and (95% CIs)	*p* value
US size	Triple negative	0.64 (0.56–0.72)	0.008
	HER-2 positive	0.65 (0.56–0.73)	0.005
	Luminal	0.74 (0.71–0.77)	< 0.001
Stiffness at SWE	Triple negative	0.55 (0.47–0.63)	0.8
	HER-2 positive	0.63 (0.55–0.71)	0.02
	Luminal	0.69 (0.64–0.70)	< 0.001

**Table 3:** Association between categorical US features and breast cancer specific survival in women by breast cancer subtype

US feature	Subtype	Hazard ratio (95% CIs)	*p* value
Skin thickening	Triple negative	4.45 (1.9–10.0)	< 0.001
	HER-2 positive	2.3 (1.1–5.0)	0.04
	Luminal	23.6 (9.1–61.3)	< 0.001
Posterior echo enhancement	Triple negative	2.2 (1.11–4.31)	0.02
	HER-2 positive	0.55 (0.22–1.39)	0.2
	Luminal	1.1(0.45–2.63)	0.84

**Fig. 3:**
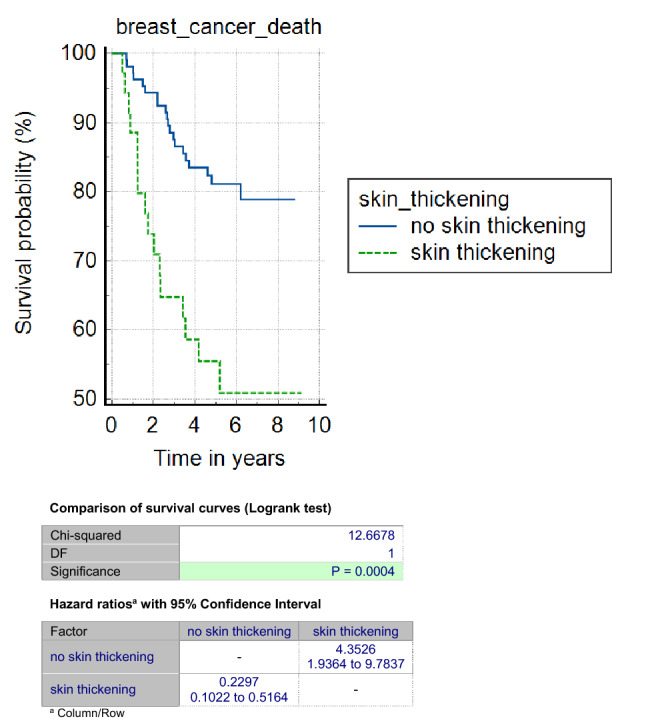
Kaplan–Meier survival curves (in years) for triple negative breast cancers according to the presence of skin thickening

US size was significantly associated with BCSS in all groups but most strongly in luminal cancers (AUC 0.74, *p* < 0.001 compared to 0.64, *p* = 0.008 for TN and 0.65, *p* = 0.005 for HER2 + ve cancers). US skin thickening was also poorly prognostic in all groups but most strongly in TN cancers (53% vs. 80% 6 year survival, *p* = 0.0004, HR 4.45 (95% CI’s 1.9–10.0), Fig. 3) and less so for HER2 + ve (66% vs. 83% 6 year survival, *p* = 0.04, HR 2.3 (95% CI’s 1.1–5.0)) and luminal cancers (80% vs. 95% 6 year survival *p* < 0.0001, HR 23.6 (95% CI’s 9.1–61.3).

**Fig. 4:**
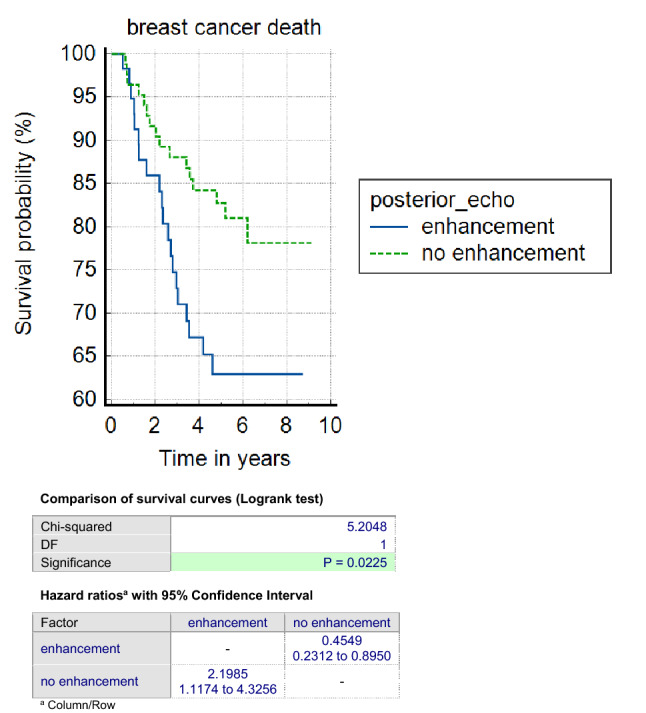
Kaplan–Meier survival curves (in years) for triple negative breast cancers according to the presence of posterior echo enhancement

Posterior echo enhancement was associated with a poor BCSS in TN cancers (63% vs. 82% 6 year survival, *p* = 0.02, HR 2.2 (95% CI’s 1.11–4.31)) (Fig. 4). Posterior echo enhancement was not associated with a poor BCSS in luminal and HER2 + ve cancers (93% vs. 95% 6 year survival, *p* = 0.84 and 78% vs. 88% 6 year survival, *p* = 0.2, respectively). The assessment of a subset of 154 patients regarding the presence or absence of posterior echo enhancement by two independent radiologists showed substantial agreement (Kappa statistic 0.66, 95% CI 0.53–0.79).

Mean stiffness at SWE was prognostic in the luminal group (6 year survival for soft vs stiff tumours was 97% vs. 89%, *p* = 0.0002, area under ROC curve 0.69, *p* < 0.001). Mean stiffness at SWE was prognostic in the HER2 + ve patients when continuous data was analysed (area under ROC curve 0.63, *p* = 0.02) but not in triple negative patients (68% vs. 80% 6 year survival, *p* = 0.15, respectively, area under ROC curve 0.55, *p* = 0.8).

The sub-analysis of patients with TN cancers who underwent NACT (*n* = 47) showed a strong relationship between RCB score and survival (area under ROC curve 0.74, *p* = 0.002). No relationship was seen between RCB score and either US skin thickening or posterior echo enhancement (area under ROC curve 0.52, *p* = 0.8 and 0.52. *p* = 0.8).

## Discussion

We have found associations between the US features of breast cancer and BCSS in all three main molecular subgroups of breast cancer. However, the individual features which are significant and the strength of such associations vary by molecular subtype. In TN breast cancer US detected skin thickening and posterior echo enhancement have strong associations with poorer outcome. In luminal cancers US size, skin thickening and stiffness at SWE appear most useful. In HER2 positive cancer significant but less striking associations are found with US size, skin thickening and stiffness at SWE.

Women with TN breast cancer have very varied outcomes but few clinically useful prognostic indictors. Basal phenotype cancers, which are often triple negative show little relationship between tumour size, nodal stage and outcome [13, 14]. Women with TN breast cancer are, therefore, a group where US prognostic indicators would be useful.

Skin thickening on US is usually not apparent clinically. In a previous study the vast majority of breast cancer deaths occurring in women with US skin thickening occurred in women without clinical evidence of skin involvement [4]. We do not have clinical examination findings in the patients in this study. However, only 23% of patients in this study who had US skin thickening received NACT. NACT has been the treatment of choice for clinical inflammatory cancers during the study period. It is, therefore, likely that the vast majority of patients in this study with US skin thickening did not have clinically inflammatory breast cancer.

The poor BCSS associated with US thickening appears to be related to the presence of a florid vascular and lymphatic plexus beneath the skin. The presence of lymphovascular invasion (VI), which is most frequently seen in a sub-dermal location, is a recognised prognostic factor in breast cancer [15, 16]. A number of previous studies have shown that a small tumour-to-skin distance is associated with lymph node metastasis in breast cancer [17, 18]. Why skin thickening has particular prognostic significance in TN breast cancers is not clear. We found no relationship between skin thickening and response to NACT so the mechanism would appear not to be related to sensitivity to chemotherapy.

It is known that posterior echo enhancement is found more frequently in high grade cancers, as many TNBCs are [19]. It has been claimed that cancers which show posterior echo enhancement are more cellular and have less desmoplasia than those cancers not showing posterior echo enhancement [20, 21]. The association between poor BCSS in TN cancers with posterior echo enhancement is not a function of histological grade as virtually all TN cancers are grade 3. Neither does the presence of posterior echo enhancement appear to be related to sensitivity to chemotherapy as we found no relationship between posterior echo enhancement and response in those TN cancers who were treated with NACT. The cause of the relationship between posterior echo enhancement and BCSS in TN cancer is unlikely to be solely due to necrosis. Necrotic tumours will tend have posterior echo enhancement due to the excellent transmission of US through fluid but necrosis occurs centrally, while posterior echo enhancement is seen behind the whole tumour. Necrosis has been found to be a poor prognostic factor in TN breast cancer [22].

The lack of an association between stiffness at SWE and outcome in TN cancers suggests that the mechanical properties of abnormal collagen structure and organisation are less important in TN cancers than in luminal cancers, where the association between stiffness and BCSS is strong. Recent previous studies have confirmed the good reproducibility of stiffness measured at SWE and US assessment of skin thickening [7, 8]. In this study we have also found assessment of posterior echo enhancement to be reliable with agreement between two independent radiologists in 85% of cases. The US features associated with outcome in breast cancer patients appear, therefore, to show good reproducibility and, therefore, promise in terms of clinical usefulness.

It should, however, be noted that other stromal markers of outcome such as TIL’s are associated with outcome in TN breast cancer [11]. Further research is needed on the correlation between TIL’s and US features.

The weaknesses of this study include that it is from a single centre and that some of the factors evaluated such as skin thickening and posterior echo enhancement were assessed retrospectively, albeit on images that had been prospectively collected and saved. While the overall number of cases and breast cancer deaths are large, the numbers in subgroups are more modest. The mean follow-up of 6.3 years means that a significant number of breast cancer deaths which will occur in this cohort have not yet happened, this will be particularly true in the luminal subgroup. However, the data for TN and HER2 + does have follow-up beyond the initial 5 years during which many events will occur.

The factors of definitive tumour grade and nodal status have not included in the analysis as these are only known with certainty post operatively. In contrast, US features are available for analysis in the preoperative setting.

In summary, we have found associations between the US features of breast cancer and BCSS in all the molecular subgroups of breast cancer but the associations vary according to subtype. In TN breast cancer US detected skin thickening and posterior echo enhancement have strong associations with poor outcome.
